# Computer Vision for Movement Observation and Recovery Enhancement (C-MORE): Box and Blocks Test

**DOI:** 10.3390/bioengineering13060602

**Published:** 2026-05-22

**Authors:** Jun Min Kim, Ziqiang (Joe) Zhu, Hari Venugopalan, Vicky Chan, Matthew K. Farrens, Samuel T. King, Andria J. Farrens

**Affiliations:** 1 Computer Science Department, University of California Davis, Davis, CA 95616, USA; jnmkim@ucdavis.edu (J.K.); ziqzhu@ucdavis.edu (Z.Z.); hvenugopalan@ucdavis.edu (H.V.); farrens@cs.ucdavis.edu (M.K.F.); kingst@ucdavis.edu (S.T.K.); 2 Physical Therapy, University of California Irvine, Irvine, CA 92627, USA; vchan2@uci.edu; 3 Research Institute of Children’s Hospital of Orange County, Orange, CA 92868, USA

**Keywords:** computer vision, stroke, clinical assessment, rehabilitation, upper-extremity kinematics

## Abstract

Stroke is a leading cause of chronic disability, with heterogeneous sensorimotor impairments that are not well captured by standard clinical assessments. While advanced motion capture and robotic systems provide precise measurements, they are not scalable for widespread clinical use. We developed C-MORE (Computer Vision for Movement Observation and Recovery Enhancement), a smartphone-based framework that uses computer vision and machine learning to automatically score the Box and Blocks Test (BBT) and extract quantitative kinematic metrics. The system combines hand tracking with a custom machine learning (ML) architecture to identify valid block transfers and segment task phases. We evaluated C-MORE in 7 individuals with chronic stroke and a cohort of 10 healthy adults. The system achieved 99.0% agreement with ground-truth scoring, with errors below clinically meaningful thresholds. Kinematic measures derived from the system were sensitive to stroke-related impairments, including reduced movement velocity and increased task duration in affected limbs. Exploratory analyses indicated that grasp-related metrics, particularly the ratio of grasp-to-transfer duration, were correlated with independent measures of proprioception. These findings demonstrate that smartphone-based computer vision can provide accurate, scalable assessment of upper-extremity function. C-MORE offers a practical approach for enhancing clinical evaluation and enabling more precise, individualized rehabilitation strategies.

## 1. Introduction

In medicine, advances in technologies such as DNA sequencing have made personalized treatment approaches possible, moving beyond one-size-fits-all treatments toward care tailored to an individual’s biology. We believe that stroke rehabilitation can follow a similar path by using computer vision techniques to provide objective, individualized assessments of movement that can better guide recovery strategies.

Stroke, caused by ischemic or hemorrhagic injury to the brain, affects approximately one in four adults and is a leading cause of chronic disability worldwide [1]. The extent of disruption to sensory and motor circuits varies widely across individuals, and emerging evidence suggests that baseline sensorimotor integrity shapes responsiveness to different rehabilitation strategies [2,3,4,5]. However, in routine care, commonly used clinical assessments are coarse and do not differentiate sensory from motor impairments with sufficient precision [2,5,6,7]. As a result, rehabilitation is typically tailored to overall motor deficit rather than underlying impairment profiles, contributing to variable and often suboptimal outcomes, with fewer than 50% of patients achieving meaningful functional recovery [8].

Patient-specific rehabilitation strategies have the potential to improve outcomes, particularly when guided by machine learning (ML) and AI models that predict response to therapy. However, the effectiveness of such approaches is constrained by the limited resolution of standard clinical assessments, which typically categorize impairment in broad ordinal terms (e.g., absent, partial, severe). In contrast, research-grade robotic and motion capture systems provide rich, quantitative measures of sensorimotor function, such as movement speed, smoothness, and range of motion, enabling more precise characterization of patient heterogeneity. Despite this promise, these systems are expensive, technically complex, and largely confined to specialized research settings, limiting the scale and diversity of quantitative datasets needed to develop robust predictive models for such a heterogeneous patient population.

Computer vision offers a scalable alternative by enabling quantitative kinematic analysis without the need for markers or specialized hardware. It has been successfully applied to the Fugl–Meyer Assessment and the Unified PD Rating Scale, improving assessment accuracy and consistency compared with clinician scoring [9]. In this work, we extend this approach to the Box and Block Task (BBT), a widely used measure of upper-extremity function in stroke, valued for its ease of administration and functional relevance [10]. Notably, BBT performance is strongly linked to hand proprioception, a predictor of responsiveness to hand therapy [3], and may therefore provide a practical pathway for distinguishing between sensory and motor deficits.

During the BBT, participants transfer as many blocks as possible over a wooden divider within one minute. Despite involving coordinated reaching, grasping, and release, the task is scored solely by the number of blocks transferred. Prior efforts to extract richer information from the BBT have used task-embedded sensors, depth cameras such as the Microsoft Kinect, virtual or augmented reality systems, or robotic interfaces [11,12,13,14,15,16]. While informative, these approaches depend on specialized hardware that introduces cost and maintenance concerns or require modifications to the assessment that limit the use of normative measures, creating barriers to widespread clinical adoption and interpretation.

Here, we instead use a standard smartphone camera to perform kinematic analysis during the BBT. To support clinical adoption, we aim to develop a system that automatically scores the assessment to ease clinician burden while providing the added benefit of more quantitative kinematic metrics that enhance clinical insight. Previous research identified grasping and transfer duration and movement amplitude and speed as meaningful metrics of hand and shoulder movement function [11,17]. Proprioception, which is believed to primarily impair grasping ability, may be quantifiable through similar kinematic assessment of block grasping. Previous groups report a high fidelity between markerless and standard motion capture analysis for these metrics, making them good candidates for computer vision applications [18].

We present the development of our assessment platform, which uses Computer Vision for Movement Observation and Recovery Enhancement (C-MORE) and was tested on 7 chronic stroke participants and 10 healthy individuals. We used an open-source, real-time hand tracking model (MediaPipe Hand [19]) in combination with custom machine learning (ML) to develop an algorithm to automatically score the BBT assessment and perform kinematic analysis of hand movement. We hypothesized that (1) C-MORE would be able to automatically score the BBT assessment with errors below the minimal clinically important difference (five blocks) [20,21], (2) kinematic metrics (speed, movement amplitude) would be sensitive to hemiparetic effects of stroke and limb dominance, and (3) grasping metrics would be associated with measures of finger proprioception.

## 2. Materials and Methods

**Experimental Setup**

Seven stroke survivors (59 ± 9.5 years, 2 female, 7 right-handed, 4 right-side-impaired) with chronic stroke (mean: 34.9, range: [7.2, 95.2] months post stroke, 3 ischemic) were included in our study. Participants were recruited from the U.C. Irvine stroke survivor database, regional hospitals and stroke support groups. The data were collected as part of a larger clinical trial on robotic hand therapy using the FINGER robot (registered on ClinicalTrials.Gov, NCT04818073). For this paper, we analyzed data from participants’ 1-month post-therapy visit.

An additional 10 healthy young adults (27 ± 4.3 years, 4 female, 12 right-handed) were recruited to further evaluate algorithm performance. Healthy individuals typically perform the task faster with fewer errors in block transferring compared to chronic stroke participants. To generate more variable movement patterns (e.g., block collisions, fumbling, and multiple block transfers) to provide a rigorous test of the scoring algorithm, we had participants perform the task while blindfolded.

**Figure 1:**
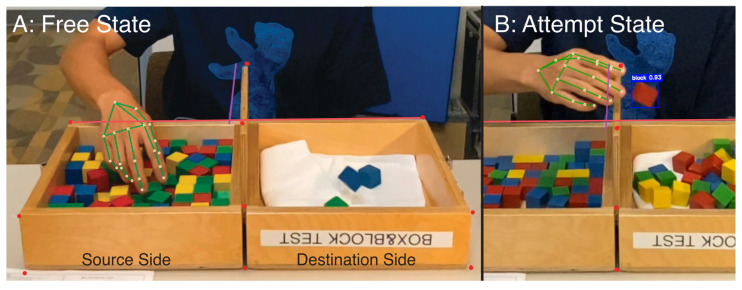
Hand detector and box detector landmarks overlaid on video stills from a participant performing the BBT. (**A**) The “free state”, during which a participant selects a block. The full test kit is shown to illustrate the 10 keypoints (red dots) on the BBT test kit identified by the Box Tracker model. The green and white dots depict the hand landmarks detected by the Hand Tracker model. The red horizontal line (source-side threshold) and the purple center line (center divider line) demark the boundaries used in the state machine to define state transitions. (**B**) The state machine is in the “attempt state” as the fingers have crossed the center line and are above the source-side threshold. The online autoscoring algorithm is shown detecting the block and its separation from the fingertips. The autoscoring algorithm logs this crossing as successful and records the finger crossing and block release timestamps.

All visits took place at U.C. Irvine, and participants gave informed consent in accordance with the UCI Institutional Review Board. All participants performed the task with their dominant (unaffected) arm first, followed by their non-dominant (affected) arm. We recorded each task at 120 FPS using multiple consumer devices (Pixel 7a, Pixel 8, Google, Mountain View, CA, USA; 9th Gen iPad, iPhone 13, Apple, Cupertino, CA, USA) mounted on a tripod for stability. No difference in recording performance was noted between devices. The field of view was centered to the midline of the assessment box and angled downward to include a view of the blocks (Figure 1).

**The Box and Blocks Test**

The Box and Blocks Test is a widely used clinical measure of gross hand dexterity and unilateral upper-limb motor function. The test kit (Performance Health, Warrenville, IL, USA) includes a rectangular box divided into two equal compartments by a divider and 150 small wooden cubes that are initially placed in the compartment closest to the limb being assessed.

All participants were given standard instructions to transfer as many blocks as possible between compartments within one minute. They were instructed to use one hand to transfer one block at a time and to cross the center divider with their hand on every transfer. The center divider of the box was aligned to the midline of the patient’s torso and set at mid-torso height.

Clinically, the assessment is scored as the total number of blocks correctly transferred. If a block bounces out of the compartment after transfer, it is still counted. If multiple blocks are transferred at once, it is scored as one block.

**Ground-Truth Scoring**

To create a set of ground-truth metrics for task scoring, we used the clinical scores recorded in person by author V.C., the physical therapist who administered the test. Additionally, each video was hand-scored by two of the authors (A.J.F., M.K.F.), who quantified the number of blocks successfully transferred as well as attempted transfers. Attempted transfers were defined as all instances in which the participant imparted a vertical motion to a block. Successful transfers, a subset of the total attempted transfers, were defined consistently with the clinical scoring described above. These scores were used to validate the computer-vision-based scoring algorithm.

To enable kinematic validation, task events were also manually annotated. Video review was used to identify grasp initiation, transfer initiation, and block release times for each transfer attempt. These times were used to delineate between the grasp (grasp initial to transfer initiation) and transfer (transfer initiation to block release) phases of task performance. Grasp onset was defined as the moment the participant first contacted a block, transfer initiation as the onset of vertical motion, and block release as the moment the block exhibited independent motion or separation from the hand.

**Online Computer Vision Scoring of the Box and Blocks Test**

To enable real-world deployment comparable to clinician-administered assessments, we designed C-MORE to perform online scoring of the BBT using off-the-shelf computer vision and a combination of custom and off-the-shelf pose estimation algorithms.

As described above, the BBT kit consists of two compartments separated by a central divider. We refer to the compartment where blocks originate as the source compartment and the compartment to which blocks must be transferred to as the destination compartment. While human administrators can readily identify transfers between these compartments, automating this process with computer vision introduces several challenges.

In this section, we outline these challenges and describe why enforcing the rules of a valid transfer is non-trivial in an algorithmic framework.

Autoscoring Architecture: To autoscore the assessment, the system tracks the coordinates of the participant’s hand and the test apparatus using two machine learning models, the Hand Tracker and the Box Tracker.

The Hand Tracker uses Mediapipe’s Hand landmark detection model to extract 21 landmarks, including the tips and joints of all fingers and the center of the wrist joint. We chose to use the MediaPipe Hand model because it is one of the most widely validated and reliable hand tracking frameworks available for real-time, real-world applications [19]. Trained on a large and diverse dataset spanning variations in hand size, skin tone, orientation, and environmental conditions, MediaPipe demonstrates strong robustness and generalizability across users and mobile device platforms [16].

The Box Tracker uses a custom-trained keypoint detection model to identify 10 landmarks that include the top four corners of the BBT test kit, the top corners and front edge of the rectangular central divider, and the front bottom left and right corners of the test kit (Figure 1). The estimated coordinate locations of the hand and the test kit are sent to the state machine to define state transitions in task performance and recorded for each frame for offline kinematic analysis. This approach enables C-MORE to calculate the precise position of the hand relative to critical boundaries such as the divider.

State Machine: We developed a state machine to support task scoring based on the hand position relative to the test kit. The system initializes in the *free state* and transitions to the *attempt state* when either (1) at least three fingertips rise above the source-side threshold or (2) any hand landmark crosses the central divider boundary.

The source-side threshold is defined as a line segment (y = mx + b) connecting the back top left and back top right keypoints of the test apparatus (Figure 1A). Similarly, the central divider line is modeled as a line segment connecting the front and back divider keypoints. Both lines are dynamically updated every 25 frames to account for any shift in position of the test kit. To account for variability in hand positioning and detection accuracy, the central divider boundary includes a buffer extending approximately 1.5 cm toward the source side, estimated based on the horizontal distance between the divider and the outer wall of the source compartment. This buffer ensures that all reasonable transfer attempts are captured (Figure 1).

For each frame, the system evaluates whether any hand landmark satisfies the attempt state logic. When the state machine enters the attempt state, the attempt logger initializes a new event record. The logging cycle concludes when the system returns to the free state.

Attempt Logger and Autoscoring Algorithm: During the attempt state, the algorithm continuously monitors the hand to determine if any hand landmark crosses the central divider line, recording the precise timestamp of the crossing in the attempt logger. In parallel, the Falling Block Detector and the Destination-Side Motion Detector, detailed below, operate to evaluate the transfer. If either detector confirms a valid release and the hand crossed the divider during the attempt, the attempt logger records a successful transfer. If the hand was not detected as crossing the divider or neither detector identified a block transfer, then the attempt logger records it as a failed attempt.

Falling Block Detector: The Falling Block Detector identifies block release events by leveraging temporal information across multiple frames, reflecting the human ability to recognize a transfer by observing a block descend from the hand over time. The method combines block detection, trajectory tracking, and heuristic validation.

Block detection was performed using a YOLO11 Nano model trained in the Ultralytics framework on ~14,000 annotated frames for 200 epochs at an input resolution of 640 × 640 pixels. During inference, candidate blocks were identified using a confidence threshold of 0.75. To reduce background noise and improve spatial resolution, detection was restricted to a dynamic region of interest derived from the hand’s bounding box with asymmetric padding, extending preferentially around the fingertips (the direction of expected block motion).

Sequential detections were linked into trajectories using an Intersection over Union (IoU)-based association criterion. A detection in the current frame is associated with an existing track if the IoU between the new bounding box and the tracked object exceeds 0.2. Detections not meeting this criterion initialized new trajectories.

To reduce false positives (e.g., stationary blocks or visual artifacts), these trajectories were filtered using three conditions. First, the minimum displacement condition required the block center to move more than 15 pixels across the trajectory. Second, the hand detachment condition required the block to show no overlap with the hand in at least one of the final three frames, ensuring separation even when the hand re-entered the region. Third, the destination validation condition required the final detected block position to lie across the central divider within the destination compartment. Trajectories satisfying all three criteria were classified as successful transfers. The time instance hand detachment was logged as the block release time in the attempt logger.

Destination-Side Motion Detector: While the Falling Block Detector is precise, it relies on tracking the block as it separates from the hand. This requirement causes failures in low-drop scenarios, where the front of the Box and Blocks Test set may occlude the release. To recover these attempts, we designed the Destination-Side Motion Detector to mimic human behavior by detecting alternative visual cues (in this case, the motion of the block(s) after it lands). Even if the fall itself is obscured, the detector identifies the transfer by spotting the block as it rolls, bounces, or settles within the destination compartment.

We trained a 3D ResNet-18 (R3D-18) Convolutional Neural Network, initialized with weights pre-trained on the Kinetics-400 action recognition dataset. For this specific application, the final fully connected layer was replaced with a single output neuron for binary classification (motion vs. static). The model was fine-tuned on a dataset of approximately 43,000 video clips, each 30 frames in length, cropped exclusively to the destination side of the box. Training utilized the Adam optimizer with a batch size of 32, employing early stopping to prevent overfitting.

When the hand crosses the central divider, the detector begins populating a rolling buffer of frames cropped to the destination-side compartment. Once the buffer accumulates 30 frames, the model performs inference to generate a motion confidence score. This sliding window analysis continues for the duration of the attempt, terminating only when the system resets for the subsequent trial. This extended observation window maximizes the likelihood of capturing a moving block. If the model outputs a confidence score exceeding 0.8 at any point during the cycle, the transfer is recorded as successful.

Model Training: To prevent data leakage between training and evaluation, we employed a strict participant-wise data split; the training subset for any given participant consisted exclusively of data from the other subjects, ensuring that no frames from the test videos appeared in the training set.

Limitations of Single-Frame Binary Classification: The dual-detector system was developed following unsuccessful initial attempts using a single-frame binary classifier to detect block release. In this approach, frames cropped to the destination compartment were passed through a model that output a probability of a block being in a falling state. In practice, this method proved unreliable due to a thresholding dilemma: lower thresholds led to excessive false positives while higher thresholds resulted in missed detections. This failure stemmed from two key limitations. First, temporal ambiguity—a single frame cannot distinguish a falling block from a stationary block without motion context. Second, lack of spatial reasoning—the model lacked explicit information about the relationship between the hand and the block, making it difficult to differentiate between grasping and release. This motivated the use of temporally and spatially informed detectors described above, in which either being true resulted in classification of a successful transfer rather than single-frame classification.

**Offline Kinematic Analysis**

Real-world Image Transformation: All keypoint detection described above was performed in pixel space. For kinematic analysis, these coordinates were transformed into real-world units and preprocessed to remove detection dropout and noise.

To transform pixels into real-world units, a homography matrix was computed using the 10 keypoints of the Box and Blocks Test apparatus and the known physical dimensions of the test kit (Figure 1). This transformation was applied to each frame to map image coordinates into a standardized coordinate system, where the front edge of the box defined the horizontal (x) axis and the central divider defined the vertical (y) axis. All coordinates were then expressed in millimeters.

The homography transformation was similarly applied to the MediaPipe-derived hand landmarks, yielding continuous positional time-series data. Missing or invalid landmark detections (e.g., points outside the workspace) were replaced with the last valid observation. The resulting signals were then screened for local outliers, defined as frame-to-frame displacements exceeding three standard deviations within a 0.25 s sliding window. Detected outliers were corrected using piecewise cubic spline interpolation.

Position data was filtered with a fourth-order zero-phase Butterworth low-pass filter (8 Hz cutoff) using filtfilt in MATLAB (R2021a). Velocity was computed as the derivative of the filtered position data and subsequently smoothed using a fourth-order zero-phase Butterworth low-pass filter (8 Hz cutoff).

Task Partitioning: Offline attempted crossing events were defined from when the fingertips came within 2.5 cm horizontally (x) and 1 cm vertically (y) of the center divider and ended when the fingertips recrossed the divider. Like the online algorithm, this lenient threshold ensured that all candidate transfers were captured, which were then labeled as successful or unsuccessful transfers by downstream logic. Each potential crossing identified offline was aligned to the online algorithm via timestamps.

For each potential crossing event, the time instance of *transfer initiation* was defined by searching backward from the crossing onset for the first local minimum in vertical hand velocity occurring once the velocity fell below 20% of the crossing velocity.

The time instance of *grasp initiation* was identified within the interval spanning from the end of the previous crossing (or start of the task) to transfer initiation. The search was restricted to potential grasp regions, defined as periods when the fingertips were below the back edge of the source compartment. Within each grasp region, grasp initiation was defined as the first local minimum in hand velocity that fell within one standard deviation of the region-specific minimum velocity. If multiple grasp regions occurred prior to a crossing (e.g., due to failed selections or dropped blocks), the region immediately preceding transfer initiation was used to define grasp initiation. Unsuccessful grasp attempts were logged for internal review only.

For successful attempts, block release times were set using the time logged by the online algorithm, determined via timestamp alignment. If the online algorithm did not identify a release time, the local maximum change (inflection point) in horizontal hand velocity was used to estimate block release.

Offline Scoring and Review Flags: For each attempted crossing event, the offline algorithm logged a finger-cross score of 1 if any hand landmark crossed over the front edge of the center divider, a score of 0.75 if the hand came within a 1 cm buffer of the divider and had normal kinematics (smooth, no reversals), and a score of 0 otherwise. Discrepancies between the offline and online finger crossing detection were flagged as “F” paired with the finger-cross score for offline review. Similarly, potential crossings with no block transfer detected were flagged “B” for review. Thus, every trial had a score (1, 0.75, 0) indicating the confidence in the fingers crossing the divider, with some trials additionally flagged with an “F” or “B” for optional review of the online algorithm detection

Offline review of flagged trials is optional and implemented to improve scoring confidence and allows clinicians to resolve ambiguous cases and provide participants with feedback on unsuccessful or borderline attempts. For example, trails flagged as “0.75-F” largely reflected instances in which the participants tossed the block over the divider and could be used to easily show participants their performance on incorrectly performed transfer attempts.

Kinematic Performance Metrics: We hypothesized that grasping and transfer phases of the BBT capture distinct aspects of proprioceptive and motor function. We calculated participants’ grasp duration as the difference between C-MORE-identified grasp initiation and transfer initiation and transfer duration as the difference between transfer initiation time and block release. C-MORE-identified events were compared with those manually annotated by human reviewers.

For kinematic analysis, we examined movement extent (sum of x and y displacement) and velocity (peak, avg) of the hand during the grasp phase and the transfer phase of movement. Hand movement was evaluated as the center of mass (COM) of the hand, defined as the average of all measured hand keypoints.

**Figure 2:**
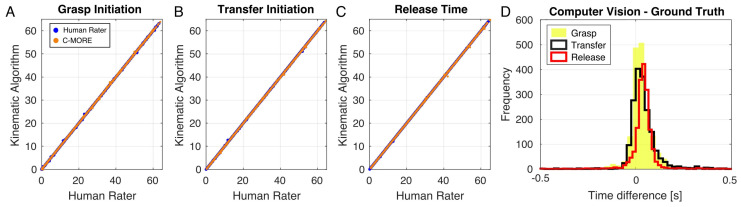
Performance of the offline algorithm in detecting (**A**) grasp initiation times, (**B**) transfer initiation times, and (**C**) release times compared to human raters and (**D**) a histogram of the difference between the computer vision identification and ground-truth evaluation used to determine any bias in event detection.

Proprioception: For the stroke cohort, we assessed participants’ proprioception using two robotic proprioception assessments (Figure 2). In the first task, Crisscross [3,22,23,24,25], the FINGER robot [26,27], crosses the index and middle fingers of the affected hand in an alternating pattern with vision of the hand occluded. The fingers were crossed between 12–54 degrees of metacarpal phalangeal (MCP) flexion. Crisscross had 20 total crossings that occurred at 10 pseudorandomized speeds between 8–18 deg/s. Proprioception was quantified as the absolute error between the MCP finger joints at button press. Crisscross has been previously validated as a measure of proprioception that is sensitive to the presence of stroke and aging [3,22,23,24,25].

The second task, Move and Match [24,28], emulates a standard clinical joint position reproduction assessment, in which the robot moves one of the participant’s fingers (index/middle) and the participant has to move the other finger (middle/index) to track the movement. One finger was moved at 8 deg/s between 12–54 degs MCP flexion for 20–30 s before coming to a rest. Participants actively tracked the finger as it was moved and were allowed to rest as needed between tracking periods. Participants performed 5 rounds of tracking with each finger. Performance was quantified as the absolute error between MCP finger joints during tracking.

Both Crisscross and Move and Match measures have a high degree of correlation to Box and Blocks Test performance [6,28]—presumably because proprioceptive deficits affect hand dexterity—and Crisscross has previously been shown to be predictive of therapy outcomes (change in Box and Blocks Test results) following robotic therapy [3,5,23]. Here, we aimed to better understand the relationship between Box and Blocks Test performance and proprioception.

**What is C-MORE’s Impact on System Performance?**

Finally, we evaluated C-MORE’s impact on system performance. Evaluating mobile system performance is important since we envision C-MORE being deployed on commodity mobile devices such as iPhones or Android devices. We focus our evaluation on C-MORE’s impact on CPU consumption, memory consumption, and power consumption.

We first ran an experiment to establish baseline metrics for CPU, memory, and power consumption by running the C-MORE’s app on a modern iPhone (iPhone 15) for one minute while disabling C-MORE’s ML pipeline. While establishing this baseline, C-MORE’s app merely rendered frames from the camera onto the iPhone’s display and wrote them to disk. We then ran another experiment to capture these metrics on the same iPhone when running all of C-MORE’s ML models to count the number of blocks transferred. To ensure that C-MORE’s ML models triggered while capturing these metrics, authors Z.Z. and J.K. performed the Box and Blocks Test in front of the iPhone’s camera. We augmented Apple Xcode’s profiler [29] with the Time Profiler instrument [30], Allocations instrument [31], and Power Profiler instrument [32] to respectively capture CPU, memory, and power consumption while running our experiments.

**Statistical Analysis**

To validate our results, we quantified the percentage accuracy and the maximum error in the number of blocks scored by C-MORE compared to the ground-truth scoring. Ground-truth attempts and C-MORE attempts were matched based on divider crossing timestamps, and the resulting correspondences were used to calculate performance metrics. Precision, recall, and F1-score were calculated using standard definitions [33].

Briefly, precision is calculated using the equation true positive/(true positives + false positives), reflecting the accuracy of detected positive block transfers. Recall is calculated as true positives/(true positives + false negatives) and reflects the system’s ability to find all the successful attempts. The F1-score is the harmonic of both precision and recall, calculated as 2 × (precision × recall)/(precision + recall). For BBT, a performance variation of 2 blocks is within the expected test–retest variability [34], and a difference of 6 blocks is the minimal clinically important difference (MCID) detectable by the assessment [21]; as such, maximum errors must be 5 or less and would ideally be between 0 and 2 blocks. 

To establish the reliability of C-MORE-based metrics compared to human raters, we calculated the intraclass correlation (ICC) of online scoring and offline scoring and grasp, transfer, and release times between C-MORE and ground-truth values. We further performed a paired *t*-test between the calculated grasp and transfer durations calculated with C-MORE compared to human evaluators to identify any systematic biases.

Kinematics measured by C-MORE were compared using *t*-tests to previously reported data in the literature [34,35,36]. To determine the sensitivity of these metrics to the effects of stroke and handedness, we used Wilcoxon’s paired signed rank testing for paired comparisons between limbs and Wilcoxon’s rank sum testing to compare performance between groups. For both groups, we expected the non-dominant/affected limb to show reduced performance (e.g., speed) compared to the other limb.

Finally, we performed an exploratory analysis of the relationship between C-MORE metrics and proprioception to determine if a sub-component of the task (e.g., grasping) showed a higher relationship to proprioception compared to the gross number of blocks transferred. To test the relationship between proprioception and performance in BBT, we performed an exploratory Spearman correlational analysis between grasping and transfer phases of BBT and proprioception.

## 3. Results

**Automatic Scoring Performance**

Table 1 summarizes the online and offline scoring and C-MORE system’s scoring algorithm across the 14 videos from the stroke cohort and the 20 videos from the unaffected adult cohort.

Stroke: For the stroke participants, out of 630 ground-truth transfer attempts, C-MORE correctly identified 625, yielding an overall accuracy of 99.0% (Table 1). Nine videos achieved perfect agreement with the ground truth (F1 = 1.000), while the remaining five videos exhibited minor discrepancies (two false positives, five false negatives).

Unaffected Adults: To evaluate generalizability, the system was tested on healthy participants performing the task under blindfolded conditions. Despite increased task speed and variability, the system maintained strong autoscoring performance. All 20 videos exhibited errors of two blocks or fewer, a range considered clinically acceptable (Table 1). Twelve videos achieved perfect agreement with the ground truth.

Overall Performance and the Role of Offline Review: Across all videos, the online scoring system achieved excellent agreement with ground-truth scoring (ICC = 0.9984, 95% CI [0.9968, 0.9992]). For comparison, a prior study that reported inter-rater reliability for human scoring of the BBT reported an ICC = 0.993 across 56 assessments. While our study was performed on a separate, smaller cohort (34 total assessments), these results suggest that the system performs comparably to or better than human assessors [34].

Manual review of the seven discrepancies in the stroke cohort revealed that one error arose from a human annotation mistake, in which the algorithm correctly identified a valid transfer that was missed by the annotator. The remaining six discrepancies were due to ambiguity in determining whether the participant’s hand crossed the divider rather than failures in detecting block transfer events. For unaffected adults, of the 16 total errors observed, only half were attributable to algorithm mischaracterization. The remaining errors resulted from divider crossing ambiguity or human annotation inconsistencies.

Importantly, for both cohorts, the system consistently detected all transfer attempts, and the offline review algorithm flagged all but one unsuccessful or ambiguous trials that required review. Trial flagging most frequently detected failed finger crossings (e.g., blocks tossed over the divider) and cases of visual ambiguity (e.g., clothing matching block color) that impacted block detection. With the inclusion of the flagging mechanism, performance reached 100% across all participants except for a single edge case in which a block balanced on the divider before returning to the source side (Table 1). For most runs, the flagging algorithm identified 0–2 trials, which would require less time to manually review than the amount of time spent standardly counting the blocks after the assessment. Cases in which greater than three blocks were flagged all corresponded to ambiguous finger crossing trials, which warranted review and possible feedback to the participant regarding task performance.

These findings indicate that the core block transfer detection pipeline operates with near-perfect accuracy and that residual errors are primarily attributable to ambiguity in enforcing task rules rather than detection limitations.

**Offline Kinematic Analysis**

Accuracy of Event Identification: The offline kinematic pipeline demonstrated excellent agreement with human raters in identifying task events (Figure 2A–C). Intraclass correlation coefficients (ICCs) exceeded 0.998 for grasp onset, 0.9999 for transfer initiation, and 0.9999 for block release across all datasets.

**Figure 3:**
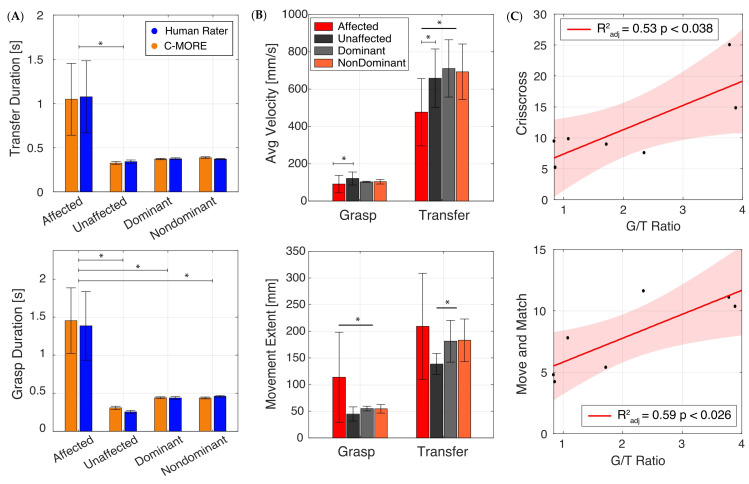
Quantitative metrics of movement performance. Asterisks denote significance at *p* < 0.05. (**A**) The estimated grasp and transfer duration for the stroke and unaffected adult groups. (**B**) The kinematic metrics derived from MediaPipe pose estimation in the two task phases of interest for the stroke and unaffected adults. (**C**) The relationship between the GTR, a proxy measure of the grasp-to-transfer duration developed to better isolate sensory deficits, and independent robotic assessments of proprioception. The GTR measure strongly correlates with both measures of proprioception.

Differences in event detection showed a small, but consistent, bias across all events of 37 ms ± 95 ms (Figure 2D). However, because this bias was consistent, differences between algorithm- and human-derived grasp and transfer durations were not statistically significant in either the stroke or healthy cohorts (Figure 3A), indicating no systematic bias in duration detection.

Comparison with Gold-Standard Metrics: Kinematic measures obtained from C-MORE were consistent with values reported in a prior motion capture study of unaffected adults performing the modified BBT [36], which has shown limited kinematic differences with the standard BBT assessment [17]. For healthy participants, mean horizontal and vertical movement ranges (258.39 ± 49.76 mm and 210.82 ± 11.83 mm, respectively) closely matched previously reported values (250.1 ± 43.1 mm and 211.4 ± 21.0 mm) [36].

Peak velocities (887.65 ± 299.2 mm/s horizontally, 1004. ± 171.4 mm/s vertically) were lower than those reported in previous motion capture studies (1431 ± 297 mm/s horizontally, 1492 ± 256 mm/s vertically) but remained within expected ranges for reach-to-grasp movements. The lower velocity in our healthy cohort was likely influenced by participants performing the assessment blindfolded.

These results support the validity of the system’s kinematic measurements despite using markerless smartphone-based tracking, although further direct validation will be performed in the future.

Sensitivity to Limb Differences: To evaluate sensitivity to motor impairment, kinematic measures were compared between affected and unaffected limbs in stroke participants.

The affected limb exhibited significantly reduced velocity during both grasp (29.5 ± 19 mm/s, *p* < 0.031) and transfer phases (182.5 ± 121.7 mm/s, *p* < 0.031) compared to the unaffected limb (Table 2, Figure 3B). Grasp and transfer durations were also significantly increased (1143 ± 1613 ms, *p* < 0.047; 721 ± 1497 ms, *p* < 0.031, respectively), consistent with known motor slowing after stroke (Figure 3A). Movement extent showed a trend toward larger values in the affected limb, suggesting reduced movement efficiency. Compared to the healthy adult participants, the affected limb showed increased movement extent, reduced transfer velocity, and increased grasp duration, consistent with known effects of stroke (Figure 3B).

In healthy participants, differences between dominant and non-dominant limbs were minimal. Only peak transfer velocity showed a non-significant trend (68.11 ± 119.9 ms, *p* < 0.13) favoring the dominant limb, while all other kinematic and temporal measures were similar across limbs.

At the individual level, stroke participants exhibited heterogeneous impairments between limbs. Most individuals showed significant (paired *t*-test, *p* < 0.05) increased movement extent (5/7) and reduced velocity (5–6/7) in the affected limb, consistent with reduced efficiency and weakness in the affected limb (Table 2). In contrast, limb differences in the healthy cohort were inconsistent and minimal, again primarily identifying increased transfer velocity of the dominant limb. 

Relationship to Proprioception: We performed an exploratory analysis to determine whether kinematic measures may reflect sensory deficits using correlational and linear regression models to test for significant relationships between kinematic measures and robotic measures of proprioception.

The BBT score showed weak-to-moderate associations with proprioception measures (BBT~Crisscross: r = −0.18, *p* = 0.71; BBT~Move and Match: r = −0.64, *p* = 0.13), following the established pattern from the literature that a greater BBT score is associated with lower proprioceptive errors. Among kinematic features, grasp-phase metrics (movement extent, grasp duration) demonstrated trend-level associations with proprioception (Crisscross: *R*_*adj*_^2^ = 0.18–0.26, *p* < 0.19; Move and Match: *R*_*adj*_^2^ = 0.33–0.35, *p* < 0.11), while transfer-phase metrics showed no associations for any measure.

We hypothesize that movement ability affects both grasping and transfer performance, while proprioception primarily affects grasping. To control for the impact of movement ability on grasping performance, we normalized each participant’s grasp duration by their transfer duration to create a grasp-to-transfer ratio (GTR). In evaluation of this exploratory measure, the GTR showed the strongest relationship with proprioceptive error (Crisscross: *R*_*adj*_^2^ = 0.53, *p* = 0.038; Move and Match: *R*_*adj*_^2^ = 0.59, *p* = 0.026, Figure 3C). Moreover, all chronic stroke individuals with significantly greater GTR compared to the healthy controls (3 std above the mean) were independently identified as having proprioceptive deficits via the Crisscross and Move and Match assessments with respect to age-matched healthy controls [6,23].

These findings suggest that task phase decomposition provides additional sensitivity to sensory impairments beyond standard BBT scoring.

**Real World Application**

**Figure 4:**
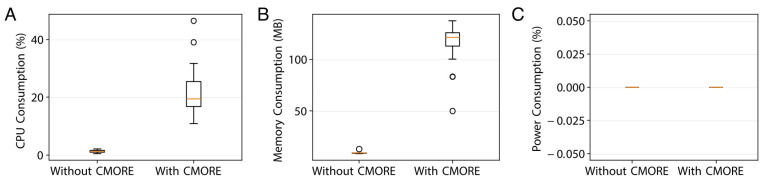
These three plots show the change in (**A**) CPU consumption, (**B**) memory consumption and (**C**) power consumption when running C-MORE on an iPhone 15 device.

Incorporating the full C-MORE pipeline increased CPU usage from 1.31% to 22.27% and memory usage from 9.52 MB to 114.56 MB, while power consumption remained negligible (Figure 4). Thus, C-MORE’s computational demands are comparable to prior mobile vision systems and support real-time deployment feasibility [37].

## 4. Discussion

In this study, we developed and evaluated C-MORE, a smartphone-based computer vision framework for automated scoring and kinematic analysis of the Box and Blocks Test (BBT). The system achieved near-perfect agreement with human evaluators while providing additional quantitative insight into movement quality, demonstrating potential for low-cost, widely available devices to be used for clinically meaningful assessment of upper-extremity function.

**Automated Scoring Performance**

C-MORE demonstrated scoring reliability comparable to human inter-rater reliability in BBT scoring (ICC > 0.998) [34]. Importantly, all observed errors were attributable to divider crossing ambiguity, a component of the assessment that is inherently difficult to standardize even among clinicians. Excluding these ambiguities, the system achieved perfect agreement with the ground truth.

This result highlights a key insight: the primary challenge in automating the BBT lies not in detecting physical events but in operationalizing clinical rules that are themselves subject to interpretation. To address this, C-MORE incorporates an offline flagging system to identify ambiguous trials that can be easily replayed for clinician review. Out of 1689 tested transfers, this flagging system identified every borderline case except for one, resulting in a 99.94% scoring accuracy after clinician review.

**Sensitivity to Motor Impairment and Limb Differences**

Beyond scoring, C-MORE provides quantitative kinematic metrics that are sensitive to both pathological and physiological differences in motor control. Stroke participants exhibited reduced movement velocity, increased movement duration, and trends toward decreased movement efficiency, consistent with established kinematic signatures of post-stroke impairment [38,39,40].

In contrast, healthy participants showed minimal differences between dominant and non-dominant limbs, with only slightly faster transfer speeds observed in the dominant arm. Although a dominant-limb advantage was expected, prior studies have similarly reported small or negligible inter-limb differences in BBT performance [14,34,41]. The absence of inter-limb differences may also be due to the blindfolded test condition, as prior evidence suggests that the non-dominant limb may exhibit greater control during movements performed without visual feedback [42,43,44]. Thus, removing visual feedback during the task may have reduced or obscured any advantage of the dominant limb.

Taken together, the subtle and variable limb differences observed in healthy participants suggest that the proposed measures are sensitive to pathological impairment rather than to normal inter-limb variability.

At the individual level, kinematic profiles revealed heterogeneous patterns of impairment in the affected limb among stroke participants, alongside minimal inter-limb differences in healthy individuals, particularly during the grasp phase of the task. These findings suggest that C-MORE measures may support more personalized characterization of the heterogenous functional deficits known to occur following stroke.

This sensitivity to both group-level and individual-level differences highlights the potential of the system for clinical monitoring and rehabilitation planning.

**Task Decomposition and Insights into Proprioceptive Ability**

A key contribution of this work is the demonstration that decomposing the BBT into grasp and transfer phases may provide additional insight into underlying sensorimotor processes. While standard BBT scoring showed only weak-to-moderate associations with proprioception in this cohort, grasp-related kinematic metrics demonstrated stronger relationships with independent measures of sensory function. The stronger associations observed to grasp-phase measures suggests that this portion of task performance may be particularly sensitive to proprioceptive deficits and worth independent evaluation. These findings are consistent with theoretical models of sensorimotor control, in which proprioception plays a critical role in object acquisition and manipulation [45,46].

In particular, the exploratory GTR measure (ratio of grasp duration to transfer duration) showed a strong relationship to both independent measures of proprioception, both on the group level (correlation) and on the individual level. On the individual level, every participant with a significantly elevated GTR had significant impairments in proprioception. By normalizing grasp duration (reflecting combined sensory and motor demands) by transfer duration (predominantly motor), this metric may more sensitively capture proprioceptive deficits independent of overall motor ability and warrants further investigation.

Given that proprioceptive impairments are common after stroke and influence functional outcomes [2,3,7,23], these results highlight the potential for computer-vision-based kinematic analysis to serve as an accessible surrogate measure of sensory impairment, which is otherwise difficult to quantify in routine clinical practice.

**Accuracy of Kinematic Measures**

While the system captured expected patterns of movement, these kinematic estimates are derived from 2D projections of inherently 3D motion. Prior work comparing monocular camera tracking to gold-standard motion capture has shown promising, but variable, results, with some reporting minimal errors and others suggesting that kinematic measures may be misestimated by up to ~10% [18,47,48]. Although standardized camera placement can mitigate these errors and preserve sensitivity to between-limb differences, further validation is needed to establish reliability across repeated assessments. Consequently, while our kinematic measures fell within expected ranges reported from previous works, it is worth noting that these measures require further validation against gold-standard marker-based systems.

In contrast, time-based measures (e.g., grasp and transfer duration) are less sensitive to these limitations and demonstrated excellent agreement with human annotation. Notably, manual identification of these events required over one hour per video, highlighting the impracticality of routine human scoring. These time-based measures not only distinguished between limbs in stroke participants but also showed unique associations with proprioceptive function.

Together, these findings suggest that while spatial kinematic measures require further validation, timing-based metrics presented here provide a reliable and validated measure that extends the traditional BBT scoring by offering insight into the underlying sources of functional impairment.

**Technical Contributions and Future Directions**

A key technical contribution of this work is the transition from single-frame classification approach to the use of a dual-detector system, which was critical for achieving robust performance. By incorporating both spatial reasoning and temporal context, the system overcomes key limitations of prior vision-based approaches that rely on static image classification [49]. However, in its current form, the “object separation” proxy still relies on frame-by-frame object detection, which lacks inherent identity persistence, allowing for edge cases where background clutter may create false positives. Since standard object detection does not guarantee that the block detected in Frame N is the same physical object as in Frame N-1, the system is susceptible to these identity swaps.

To mitigate this, future work will focus on integrating object tracking algorithms (e.g., Kalman filters or optical flow) rather than pure object detection. By maintaining a temporal history of each block’s trajectory, the system can enforce a “region of interest” (ROI) prediction and restrict the search space for the next frame based on the block location in the prior frame. This would significantly reduce the likelihood of locking onto background distractors and improve processing speed by ignoring irrelevant regions of the image.

To further harden the system against errors, we plan to move from a dual-proxy (OR logic) system to a triple-proxy consensus (voting) system. This voting mechanism would avoid the problem that when two proxies disagree, which should we trust? In this model, a successful block drop would be confirmed by a majority vote (two out of three), drastically reducing false positives. Accomplishing this will require adding a third independent validation method—currently we plan the third method to identify when the hand contains a block while crossing the divider and is empty upon return.

As mentioned previously, another source of error in assessment scoring is accurate identification of divider crossing, which had the greatest ambiguity in our evaluation. While we have addressed this issue by providing offline trial flagging, we have additionally added a real-time feedback option. The C-MORE app now has a real-time feature that can provide auditory feedback—a short beep—when it registers that the hand has crossed the divider. This gives participants immediate confirmation of whether an attempt was counted, reducing ambiguity during test administration.

Finally, a primary limitation of the current implementation is the reliance on 2D pixel coordinates to define algorithmic state transitions. While effective for a wide range of camera angles, this approach degrades if the camera is positioned at a steep angle (e.g., high up and looking down). Our current application addresses this issue by providing an on-screen outline of the Box and Blocks Test kit for users to align the camera to, similar to the guidance provided by online mobile banking apps for check alignment. Future iterations will attempt to resolve this by fully leveraging the box keypoints to establish a 3D coordinate system, allowing us to define planes in 3D space that are invariant to camera perspective.

**Accessibility, Scalability and Clinical Translation**

A major advantage of C-MORE is its accessibility. Unlike prior approaches that use specialized hardware and infrastructure (sensorized blocks, robotic systems, VR, AR, Microsoft Kinect) [11,12,13,14,15], C-MORE uses a commodity smartphone and the standard test kit, which enables scalable deployment in clinical and potentially home environments.

Our framework was specifically designed for real-time hand tracking and deployment across a wide range of mobile devices. We tested our platform using a variety of phablet devices, including Android and Apple products. Our app showed no meaningful difference in performance regardless of device, as long as the device could acquire a 120 FPS recording. We selected MediaPipe Hand for pose estimation as it provides robust real-time tracking of individual digits and strong performance across variations in hand size, skin tone, orientation, and environmental conditions. Its computational efficiency and cross-platform compatibility support practical clinical translation. This accessibility has important implications for increasing the frequency and granularity of patient monitoring, thereby supporting more personalized and adaptive rehabilitation strategies [50].

Compared to other markerless motion capture assessments of BBT [12,51], the C-MORE system has higher accuracy in scoring and delineation of grasping and transfer task phases and integrates seamlessly into existing workflows [12,51]. Although VR- and AR-based systems are promising because they similarly leverage commercially available hardware and uniquely support assessment without the physical test kit, these approaches modify standard BBT administration by altering the test interface and removing tactile sensory feedback during block interactions. Consequently, these systems have reported differences in block transfer counts relative to conventional BBT administration, limiting comparability with established normative datasets [11,15,16]. Furthermore, the absence of tactile and object-interaction feedback in these assessments may reduce sensitivity of these assessments to proprioceptive impairments that contribute to dexterous hand function. In contrast, C-MORE integrates markerless motion capture directly into the standard assessment workflow, enabling extraction of detailed kinematic measures while preserving conventional testing procedures. Our system aligns with broader efforts to automate clinical motor assessments, such as the Fugl–Meyer Assessment, that have shown that computer vision can improve assessment objectivity and reproducibility [9].

Finally, although these measures were designed to provide greater clinical insight, they may also offer value for patient feedback and motivation. With C-MORE (Supplementary Video S1), participants can observe improvements in performance—such as reduced grasp time or increased transfer speed—even when these changes may not immediately translate to a higher block count. This may help reinforce progress that would otherwise go undetected by standard BBT scoring.

**Study Limitations**

While promising, our work has several limitations that should be acknowledged. First, the sample size, particularly in the stroke cohort, was relatively small, which may limit generalizability. For example, the exploratory correlational analysis between GTR is under-powered. To reliably detect a correlation of 0.76, as reported in this paper at *p* < 0.05, a power analysis indicates at least 9 participants are required, and good practice for correlational analysis recommend at least 20 participants. Thus, future studies should validate the system in larger and more diverse populations. Second, while the system performed well under semi-controlled laboratory conditions, variability in lighting, camera positioning, and background environments may impact performance in real-world settings. Similar challenges have been reported in markerless motion capture and computer vision applications in rehabilitation [9,18,47].

Finally, while the system provides rich kinematic data, the clinical interpretation of these metrics requires further investigation. Establishing normative ranges and clinically meaningful thresholds will be essential for translating these measures into actionable insights for clinicians. Integration with digital health platforms and remote monitoring systems may further enhance clinical utility and accessibility.

## 5. Conclusions

In conclusion, C-MORE represents a significant step toward scalable, objective, and quantitative assessment of upper-extremity function. By combining accurate automated scoring with detailed kinematic analysis and task decomposition, the system extends the utility of the BBT beyond outcome measurement to capture underlying sensorimotor processes. With further validation, this approach has the potential to enhance clinical decision-making, support personalized rehabilitation, and ultimately improve patient outcomes.

## Figures and Tables

**Table 1:** Table reporting the performance of the online scoring algorithm (GT-F_1_) and performance with the offline trial flagging review (“Flagged”, “Acc”) for chronic stroke participants (top) and unaffected adults (bottom).

Video Name	^a^GT	^b^Det	^c^FP	^d^FN	Prec.	Recall	F_1_	Flagged	^e^Acc
Chronic Stroke									
CONK_Affected	4	4	0	0	1.000	1.000	1.000	1	1.000
CONK_Unaffected	71	70	0	1	1.000	0.986	0.993	1	1.000
FRAP_Affected	47	48	1	0	0.979	1.000	0.989	7	1.000
FRAP_Unaffected	49	50	1	0	0.980	1.000	0.990	2	1.000
LEDU_Affected	48	48	0	0	1.000	1.000	1.000	0	1.000
LEDU_Unaffected	55	55	0	0	1.000	1.000	1.000	2	1.000
MASR_Affected	54	54	0	0	1.000	1.000	1.000	3	1.000
MASR_Unaffected	76	76	0	0	1.000	1.000	1.000	3	1.000
RUPD_Affected	3	3	0	0	1.000	1.000	1.000	2	1.000
RUPD_Unaffected	57	57	0	0	1.000	1.000	1.000	3	1.000
SARS_Affected	14	14	0	0	1.000	1.000	1.000	2	1.000
SARS_Unaffected	47	47	1	1	0.979	0.979	0.979	9	1.000
ZHAE_Affected	36	36	0	0	1.000	1.000	1.000	1	1.000
ZHAE_Unaffected	68	65	0	3	1.000	0.956	0.977	12	1.000
Unaffected Adults									
Subj_01-LeftHand	50	51	1	0	0.980	1.000	0.990	2	1.000
Subj_01-RightHand	48	48	0	0	1.000	1.000	1.000	0	1.000
Subj_02-LeftHand	49	50	1	0	0.980	1.000	0.990	0	0.990
Subj_02-RightHand	54	54	1	1	0.981	0.981	0.981	2	1.000
Subj_03-LeftHand	54	54	0	0	1.000	1.000	1.000	0	1.000
Subj_03-RightHand	51	52	1	0	0.981	1.000	0.990	2	1.000
Subj_04-LeftHand	50	51	1	0	0.980	1.000	0.990	1	1.000
Subj_04-RightHand	51	51	0	0	1.000	1.000	1.000	0	1.000
Subj_05-LeftHand	57	57	0	0	1.000	1.000	1.000	0	1.000
Subj_05-RightHand	56	56	0	0	1.000	1.000	1.000	0	1.000
Subj_06-LeftHand	60	60	0	0	1.000	1.000	1.000	0	1.000
Subj_06-RightHand	52	52	0	0	1.000	1.000	1.000	2	1.000
Subj_07-LeftHand	56	56	0	0	1.000	1.000	1.000	0	1.000
Subj_07-RightHand	51	51	0	0	1.000	1.000	1.000	0	1.000
Subj_08-LeftHand	53	53	0	0	1.000	1.000	1.000	0	1.000
Subj_08-RightHand	56	56	0	0	1.000	1.000	1.000	4	1.000
Subj_09-LeftHand	54	54	0	0	1.000	1.000	1.000	1	1.000
Subj_09-RightHand	49	51	2	0	0.961	1.000	0.980	1	1.000
Subj_10-LeftHand	54	52	1	3	0.981	0.944	0.962	5	1.000
Subj_10-RightHand	55	54	0	1	1.000	0.982	0.991	3	1.000

^a^GT = Ground truth block transfer counts; ^b^Det = Detected block transfer counts; ^c^FP = False positive; ^d^FN = False negative; Prec. = Precision; Flagged = Trials flagged by offline algorithm; ^e^Acc= Accuracy after reviewing flagged trials.

**Table 2:** Table reporting the numerical differences between groups for the quantitative metrics of movement performance plotted in Figure 3A,B. Group-level differences report the significance of Wilcoxon’s paired *t*-testing, and significant differences are bolded. The individual-level differences report the percentage of individuals within each group that had significant (Wilcoxon’s two-sample *t*-test, *p* < 0.05) differences between limbs.

	Group Level Limb Differences				Individual Subject level Difference			
Task Phase	Stroke (U:A)		Healthy (D:N)		Stroke		Healthy	
Grasp	Mean (Std) ms	Sig	Mean (Std)	Sig	Num U > A	Num U < A	Num D > N	Num D < N
Duration	1143 (1613)	***p* < 0.047**	14 (48)	*p* = 0.492	0.00%	57.14%	0.00%	0.00%
Extent	−72.37 (100.91)	*p* < 0.078	0.14 (6.61)	*p* = 1.0	14.29%	71.43%	10.00%	0.00%
Peak Vel	38.87 (55.52)	*p* < 0.156	−1.82 (24.69)	*p* = 1.0	57.14%	0.00%	0.00%	10.00%
Average vel	29.54 (19.34)	***p* < 0.031**	−0.68 (12.22)	*p* = 0.77	85.71%	0.00%	0.00%	10.00%
Transfer	Mean (Std) ms	Sig	Mean (Std)	Sig	Num U > A	Num U < A	Num D > N	Num D < N
Duration	721 (1497)	***p* < 0.031**	3 (43)	*p* = 0.922	0.00%	85.71%	20.00%	30.00%
Extent	−73.51 (92.58)	*p* < 0.078	−1.76 (22.23)	*p* = 0.56	28.57%	71.43%	20.00%	10.00%
Peak Vel	200.16 (174.41)	***p* < 0.047**	68.11 (119.85)	*p* < 0.131	71.43%	14.29%	30.00%	10.00%
Average vel	182.54 (121.73)	***p* < 0.031**	18.26 (61.73)	*p* < 0.32	71.43%	0.00%	40.00%	20.00%

## Data Availability

Data will be made available upon reasonable request to the corresponding author, Andria J. Farrens.

## Supplementary Materials

The following supporting information can be downloaded at: https://www.mdpi.com/article/10.3390/bioengineering13060602/s1, Video S1: Live Demonstration of the C-MORE App.

## Abbreviations

The following abbreviations are used in this manuscript:

BBT	Box and Blocks Test
ML	Machine Learning
GTR	Grasp-to-Transfer Ratio
C-MORE	Computer Vision for Movement Observation and Recovery Enhancement
FINGER	Finger INdividuating Grasp Exercise Robot
